# Studies on the Efficiency of Iron Release from Fe(III)-EDTA and Fe(III)-Cit and the Suitability of These Compounds for Tetracycline Degradation

**DOI:** 10.3390/molecules27238498

**Published:** 2022-12-02

**Authors:** Agnieszka I. Piotrowicz-Cieślak, Maciej Maciejczyk, Małgorzata Margas, Dariusz Rydzyński, Hanna Grajek, Dariusz J. Michalczyk, Janusz Wasilewski, Bogdan Smyk

**Affiliations:** 1 Department of Plant Physiology, Genetics and Biotechnology, Faculty of Biology and Biotechnology, University of Warmia and Mazury in Olsztyn, Oczapowskiego 1A, 10-718 Olsztyn, Poland; 2 Department of Physics and Biophysics, Faculty of Food Science, University of Warmia and Mazury in Olsztyn, Oczapowskiego 4, 10-719 Olsztyn, Poland; 3 Department of Biochemistry, Faculty of Biology and Biotechnology, University of Warmia and Mazury in Olsztyn, Oczapowskiego 1A, 10-718 Olsztyn, Poland

**Keywords:** antibiotic elimination, absorption, fluorescence, degradation kinetics, reaction rate

## Abstract

Iron ions can be used to degrade tetracycline dispersed in nature. Studies of absorption and fluorescence spectra and quantum chemistry calculations showed that iron is more readily released from Fe(III)-citrate than from Fe(III)-EDTA, so Fe(III)-citrate (Fe(III)-Cit) is more suitable for tetracycline (TC) degradation. At 30 °C, a severe degradation of TC by Fe(III)-Cit occurred as early as after 3 days of incubation in the light, and after 5 days in the dark. In contrast, the degradation of TC by Fe(III)-EDTA proceeded very slowly in the dark. By the fifth day of incubation of TC with Fe(III)-Cit in darkness, the concentrations of the former compound dropped by 55% and 75%, at 20 °C and 30 °C, respectively. The decrease in tetracycline concentrations caused by Fe(III)-EDTA in darkness at the same temperatures was only 2% and 6%, respectively. Light increased the degradation rates of TC by Fe(III)-EDTA to 20% and 56% at 20 °C and 30 °C, respectively. The key role of the light in the degradation of tetracycline by Fe(III)-EDTA was thus demonstrated. The TC degradation reaction showed a second-order kinetics. The rate constants of Fe(III)-Cit-induced TC degradation at 20 °C and 30 °C in darkness were *k* = 4238 M^−1^day^−1^ and *k* = 11,330 M^−1^day^−1^, respectively, while for Fe(III)-EDTA were 55 M^−1^day^−1^ and 226 M^−1^day^−1^. In light, these constants were *k* = 15,440 M^−1^day^−1^ and *k* = 40,270 M^−1^day^−1^ for Fe(III)-Cit and *k* = 1012 M^−1^day^−1^ and 2050 M^−1^day^−1^ at 20 °C and 30 °C; respectively. A possible reason for the higher TC degradation rate caused by Fe(III)-Cit can be the result of its lower thermodynamical stability compared with Fe(III)-EDTA, which we confirmed with our quantum chemistry calculations. Two quantum chemistry calculations showed that the iron complex with EDTA is more stable (the free energy of the ensemble is 15.8 kcal/mol lower) than the iron complex with Cit; hence, Fe release from Fe(III)-EDTA is less effective.

## 1. Introduction

In recent years, antibiotics have been broadly applied in human and veterinary medicine, resulting in their accumulation in the environment. Unlike other more commonly known pollutants, e.g., pesticides and detergents, pharmaceuticals enter the environment in low quantities but continuously [1]. The fact that pharmaceuticals are detected, albeit in trace amounts, in aqueous ecosystems indicates that they have become a very widespread form of pollutants [2]. Most antibiotics remain stable in the environment and may persist long after they have been excreted by animals [3]. Antibiotics are neither very readily absorbed nor metabolised by animal cells, so 30% to 90% of ingested doses are released to the environment in faeces and urine, virtually unmodified or transformed into derivatives that retain most of their biological activity [4,5]. Over 400 bioactive compounds are applied as components of nearly 2000 veterinary pharmaceuticals [6,7]. In the EU, 8935 tons of veterinary antibiotics were sold in 2014, and in Poland alone, it was 581.3 t in the same year. Among all available antibacterial drugs, tetracyclines were sold most often (33.4%) [8]. Pharmaceutical pollutants are difficult to remove. Drugs may even be detected in water released from water treatment plants as the efficiency of their removal is very low [9]. The elimination of tetracyclines by water treatment with iron might prove to be the optimal solution of this problem. Iron is one of the most widespread metals on Earth, amounting to 5.1% of the Earth’s crust. Depending on environmental conditions, it may take form of bivalent or trivalent cations [10]. The redox transformations of iron play a significant role in soils and nontoxic sediments. In the presence of oxygen, iron is stable only under acidic conditions, while at neutral pH, it readily oxidises to Fe(III). Soils and sediments may contain iron amounts of up to 10 mmol/kg of dry weight, and the ferric ion is the main acceptor of electrons in soils [11,12]. The removal of tetracycline from the environment is of particular importance considering that in soil this antibiotic can retain its antibacterial properties for a fairly long time [13]. In slurry, it takes 578 days for TC degradation to reach 50% [14]. TC from water and soil can be incorporated into the trophic chains [15,16]. The treatments for TC removal from water that have been tested so far have included ozonation and the application of iron(III) sodium ethylenediaminetetraacetate, iron(III) trisglycinate, and iron(III) citrate (Fe(III)-Cit) [17], as well as TC adsorption on sorbents based on metal or metal oxides, such as montmorillonite [18,19], magnesium-aluminum hydrotalcites [20], hydrous oxides of aluminium and iron [21], iron oxides, and iron oxide-rich soils [22]. Liu et al. [23] have demonstrated high efficiency of TC removal from water using Fe-Mn binary oxide. The presence of a fair number of functional groups in the TC molecule facilitates its protonation and deprotonation, depending on pH. As a result, TC is easily chelated by metal cations, formed by Ca, Mg, Cu, Zn, Fe, Al, Cd, Co and Pb [24]. Among metal ions, the cations of Fe are the most prevalent in aqueous environments. In rivers, their concentrations range from 0.05 to 6.5 mg × L^−1^ [25]. Chen et al. [26] showed that the presence of Fe(III) ions in water can accelerate the photolysis of TC and facilitate its removal from the environment.

Fe(III)-EDTA and iron (III) citrate applications and impacts on the environment have been studied by several authors as these compounds are widely used in agriculture and are therefore commonly found in soil. In agriculture, Fe(III)-EDTA is applied for foliar treatments in solutions of 13% or 15% [27] in cultivation of rape, cereals, potatoes or beets. Additionally, in greenhouse cultivation, Fe(III)-EDTA is mainly used in vegetable and fruit production [28]. It is used to stabilise herbicides or other water-soluble or water-miscible herbicide formulations. Fe chelates are applied in various crops to eliminate leaf chlorosis [29]. Based on the type of chelates used, the agricultural market is divided into EDTA, EDDHA, DTPA, IDHA and others. The use of Fe(III)-EDTA is predicted to dominate due to its ability to bind micronutrients such as iron, calcium, zinc, manganese and copper. EDTA chelates are cheap and readily available. In Europe, most chelates in agriculture are used in Germany, the Netherlands and Norway [30]. Similar to Fe(III)-EDTA, Fe(III) citrate can be used to eliminate chlorosis in leaves [31]. Citrates are also released by plant roots into the soil, especially under stress conditions [32]. The concentration of citrate in soil can be variable and depends on soil type [33]. Ferric citrate is also used in medicine to regulate blood iron levels in patients with chronic kidney disease undergoing dialysis [34,35]. The occurrence of significant amounts of Fe(III)-EDTA and Fe(III)-Cit in soil may contribute to the removal of tetracycline from the environment.

The degradation of TC in the environment takes place under the influence of Fe ions released from molecular complexes such as Fe(III)-EDTA and Fe(III)-Cit. Iron attaches to tetracycline and degrades it. In our earlier studies [36], we showed that Fe(III) ions attach to the BCD ring and to the A ring of tetracycline, and other divalent compounds such as Mg^2+^ [37] attach to form complexes with tetracycline. Rydzyński et al. [37] showed that tetracycline has a high affinity for divalent metals and readily extracts magnesium even from molecule chlorophyll. Therefore, the goal of the present study was to determine the difference in the speed of the release of iron from Fe(III)-EDTA and Fe(III)-Cit complexes and thereby point out which of these compounds should be more effective in degrading tetracycline in the environment. Both compounds, Fe(III)-EDTA and Fe(III)-Cit, are introduced into the soil in large quantities by humans [27,28,29,30,31,32,33,34,35], so using them to remove tetracycline would likely pose no significant environmental burden. It should be emphasized that TC is a new environmental pollutant (significant amounts of this antibiotic in soils [38] and waters [39]) causing, among other things, the destruction of chlorophyll in plants [40]; plant dysfunction and the inhibition of photosynthesis [41], growth and the activity of many enzymes [42].

Light and temperature are the main factors affecting many chemical reactions under typical environmental conditions. Therefore, the aim of our work was to determine which of the compounds, Fe(III)-EDTA or Fe(III)-Cit, release iron more readily, how this process depends on light and temperature, and whether it results in any appreciable degradation of tetracycline. The rate of tetracycline degradation was monitored spectroscopically, and the free energy of binding of Fe(III) to Cit and EDTA molecules was calculated using quantum chemistry methods.

## 2. Results and Discussion

In order to the rate of iron release from Fe(III)-EDTA and Fe(III)-Cit and the resulting TC degradation by iron from these compounds, the absorption and fluorescence spectra of tetracycline solutions with Fe(III)-EDTA or Fe(III)-Cit were measured.

### 2.1. Absorption Spectra

**Figure 1:**
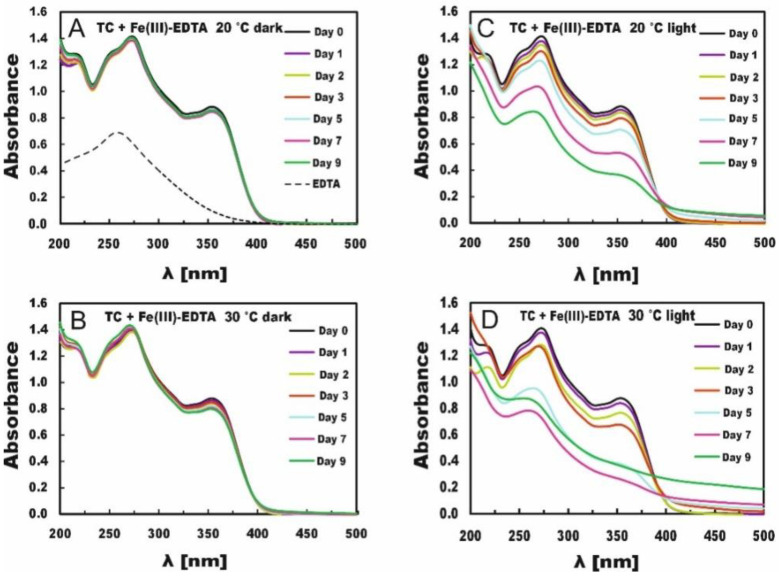
Changes in absorption spectra of TC in the presence of Fe(III)-EDTA. The samples were stored in the dark (**A**,**B**) or in light (**C**,**D**) at 20 °C (**A**,**C**) and 30 °C (**B**,**D**).

**Figure 2:**
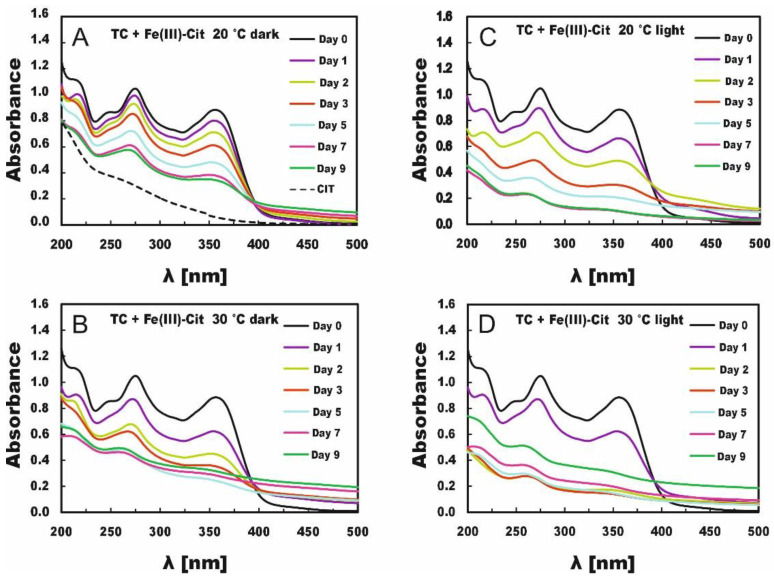
Changes in TC absorption spectra under the influence of Fe(III)-Cit in solutions stored in the dark (**A**,**B**) or in light (**C**,**D**) at 20 °C (**A**,**C**) and 30 °C (**B**,**D**).

The measurements of the absorption spectra were carried out within 9 days, starting one hour after mixing solution’s components (day 0) and then after 1, 2, 3, 5, 7 and 9 days. The changes in TC absorption spectra proceeding over 9 days are presented in Figure 1 for TC with Fe(III)-EDTA and in Figure 2 for TC with Fe(III)-Cit.

The changes in the absorption spectra of the TC solutions with Fe(III)-EDTA stored in the dark at both 20 °C and 30 °C were negligible (Figure 1A,B), which indicates that Fe (III) was released very slowly from this compound in the dark, especially at 20 °C. On day 9 of measurements at temperature 20 °C, the absorbance at the maximum (λ = 353 nm) absorption band dropped from A = 0.884 only to A = 0.854, whereas at 30 °C, it dropped to A = 0.800. That is, increasing the temperature resulted only in a slightly faster release of iron needed for the degradation of tetracycline. The action of light, however, accelerated the process of iron release and resulted in larger changes in the absorption spectra.

At 20 °C, on day 9 of measurement in the light conditions, the absorbance dropped to A = 0.363 (Figure 1C). However, as soon as after 7 days, slight turbidity of the solution could be observed, manifested by absorbance increases at wavelengths above 420 nm (compared with the results obtained for TC stored in the dark (Figure 1A), the absorption should reach zero). At 30 °C, on day 7 (Figure 1D) the absorbance dropped to A = 0.280, whereas the solution turned slightly turbid as soon as on day 5. Afterward, a sharp absorbance increase was observed on day 9 (particularly above 420 nm) due to the more intense solution turbidity and potential precipitation of iron oxides [43]. The absorption bands shown in Figure 1 indicate the predominating effect of light on TC degradation in the presence of Fe(III)-EDTA and a minor effect of temperature increase on the samples stored in the dark. Table 1 provides concentrations of TC solutions and the percentage contributions of TC concentration compared with the control in TC solutions and Fe(III)-EDTA. For the irradiated samples, no concentrations were provided for days 7 and 9 because they were turbid (probably due to the precipitation of iron oxides), their absorbance increased, and it was impossible to achieve correct concentrations. The comparison of the percentage decrease in TC concentration in the solution, e.g., on day 5 showed that it reached 2% and 6% for the TC solutions stored in the dark at 20 °C and 30 °C, respectively, as well as 20% and 56% for the TC solutions stored in light at respective temperatures.

TC degradation depends on the concentration of iron releasing from the chelator. Chen et al. [26], e.g., studied porphyrin-ligated Fe(III)-carboxylate complexes and Fe(III)-siderophore complexes. The rate of Fe release from the chemicals depends on the location of the metal binding sites within those molecules and the physical conditions of the reaction. In our experiments, light was a strong determinant of iron release from the Fe(III)-EDTA molecule. TC degradation in the dark accounted for barely 3%, which proves that Fe(III)-EDTA needed light for Fe release from the molecule. The temperature increase to 30 °C enhanced TC degradation by Fe(III)-EDTA to 7%. It is, however, still not much compared with the light-induced degradation. Therefore, light seems to be an important factor increasing efficiency of degradation of TC [26].

TC degradation in the presence of Fe(III)-Cit proceeded much faster (Figure 2), and profound changes were observed in absorption spectra even in the dark (compare with Figure 1). The absorption spectra of TC solutions with Fe(III)-Cit, including even those stored in the dark, displayed significant changes within 9 days. The absorption spectra of the solutions stored at 30 °C for just 5 days did not resemble the TC absorption spectra of fresh TC solutions. In turn, the TC absorption spectra of the light-exposed samples were deformed as early as after 3 days, which pointed to almost complete TC degradation. The temperature also strongly affected TC degradation in this case, i.e., TC concentration decreased by 66% on day 9 in the samples stored at 20 °C in the dark, whereas in those stored at 30 °C, it decreased by 75% as soon as after 5 days. The degradation process was more drastic under light conditions, reaching 79% in the samples stored at 20 °C (on day 5) and 86% in those stored at 30 °C (day 3) (Table 2). It should be emphasized that the release of Fe from Fe (III)-Cit occurs not only in the light but also in the dark.

**Figure 3:**
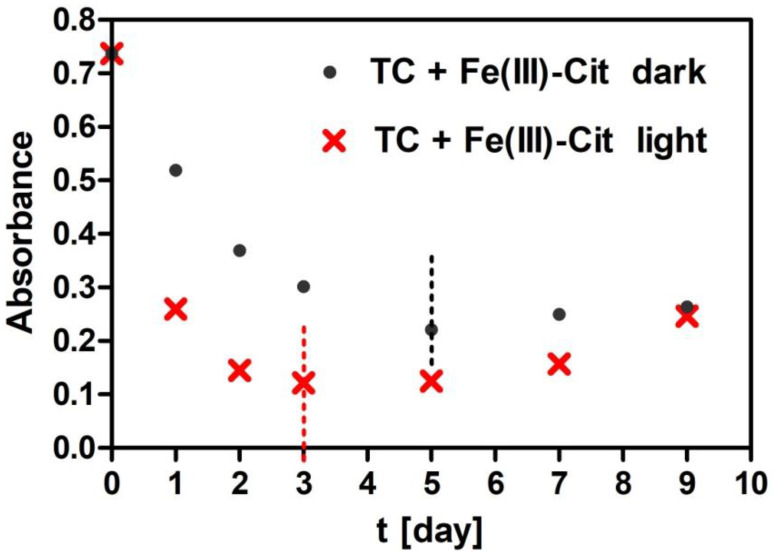
Changes in absorbance of TC + Fe(III)-Cit solutions stored at 30 °C as a function of time measured at the maximum long wavelength absorption band of TC. Dashed lines indicate intervals until which the samples were not turbid.

The comparison of TC degradation in the solutions with Fe(III)-EDTA and with Fe(III)-Cit stored under the same conditions indicates that iron is released faster from Fe (III)-Cit than Fe(III)-EDTA. The release of iron from Fe (III)-Cit occurs both in light and in the dark but from Fe(III)-EDTA only in light. Chen et al. [26] indicate that TC degradation occurs by its strong complexing with Fe(III). In our case, the deformation of the absorption spectra that appears as a result of attaching Fe (III) to TC is bigger in the presence of Cit than of EDTA in the light conditions. In EDTA under darkness conditions, it practically does not proceed at all. This is due to a poor release of iron Fe from EDTA and hence the lack of iron ions in the EDTA solution kept in darkness. Both Table 1 and Table 2 are missing concentrations of certain solutions determined on days 5, 7 and 9 (Table 2) due to their turbidity. In order to explain the lack of these concentrations for Cit, an exemplary dependence of the absorbance as a function of time for TC stored at 30 °C (for λ = 353 nm) is shown in Figure 3. In the case of samples stored in the dark, the results from days 7 and 9 and in light on days 5, 7 and 9 were unsuitable for analysis because the absorbance was increasing due to the appearance of turbidity in the solution. Therefore, those values could not be taken for calculations in the kinetic analysis.

Wang et al. [44] did not observe turbidity in any of the experiments with TC or Fe ions within 72 h. Their experiments were conducted at initial TC concentrations of 40, 80, 120, 160 and 200 mM in the presence of 40 mM Fe(III) with the spectrum recording time of 72 h. They assumed the enhanced solubility of Fe(III) in the reaction solutions resulting from the complexation of TC with Fe ions. Our experiment lasted longer, 216 h, and within 72 h, just as with Wang et al. [44], we did not observe any turbidity of the solution. Turbidity appeared in our experiment only after 168 h or 196 h, depending on the dark, light and temperature conditions of the experiment (see Table 1 and Table 2 and Figure 3). The observed drop in TC absorption elicited by Fe(III)-EDTA and Fe(III)-Cit compounds may point to the reaction of capturing ferric ions from the chelators by TC. The Fe(III) ion binding with TC has already been studied by other authors [45,46]. Saghi and Mahanpoor [47] demonstrated that nanophotocatalytic α-Fe_2_O_3_/12-TSA 7H_2_O accelerated TC degradation compared with pure α-Fe_2_O_3_NPs. In turn, Smyk et al. [36] and Wang et al. [44] showed that the TC degradation was stimulated by light. In order to study the kinetics of TC degradation by Fe(III)-EDTA and Fe(III)-Cit, the reaction rate constants were calculated.

The degradation of TC in the presence of Fe(III)-EDTA and Fe(III)-Cit was described with the second-order kinetics reaction. The dependence of TC concentration on time was described by the kinetic equation $\frac{1}{C}=\frac{1}{C_{0}}+k\left(t\right)$.

**Figure 4:**
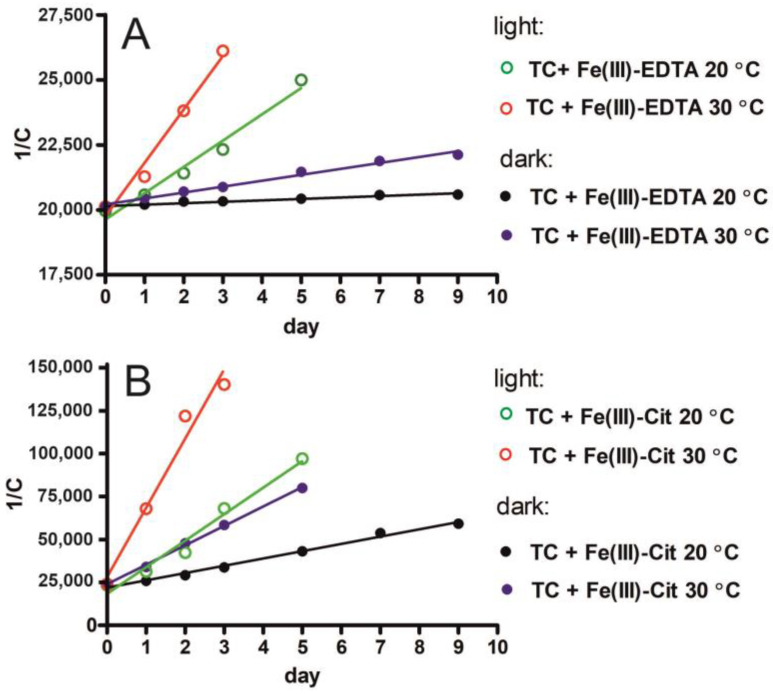
The relationship between TC concentration and time of Fe(III)-EDTA (**A**) and Fe(III)-Cit (**B**) action at various temperatures (20 °C and 30 °C) under light and dark conditions. The following equation was used: 1/C = 1/C_0_ + *k*t.

Calculations were made with the concentrations provided in Table 1 and Table 2. Figure 4 presents the $\frac{1}{C}=f\left(t\right)$ dependency for TC solutions with Fe(III)-EDTA and Fe(III)-Cit under four variants of experimental conditions: 20 °C, 30 °C, dark, and light.

The degradation rate constants (*k*) of TC with Fe(III)-EDTA and Fe(III)-Cit are provided in Table 3. In the case of TC with EDTA, *k* reached 55.4 ± 6.8 M^−1^day^−1^ and 227 ± 12 M^−1^day^−1^ for the samples stored at 20 °C and 30 °C, respectively, and were low compared with the *k* values obtained for light-induced degradation: 1012 ± 93 M^−1^day^−1^ and 2050 ± 210 M^−1^day^−1^ at 20 °C and 30 °C, respectively (Table 3). In the case of Fe(III)-Cit, *k* reached 4240 ± 180 M^−1^day^−1^, 11,330 ± 290 M^−1^day^−1^, 15,440 ± 1450 M^−1^day^−1^ and 40,270 ± 5180 M^−1^day^−1^ under respective conditions. The TC degradation rate constants obtained under various conditions of sample storage indicate that TC degradation by Fe(III)-EDTA at room temperature (20 °C) in the dark proceeded very slowly and with a very low reaction rate constant. By contrast, the degradation of TC by Fe(III)-Cit in the dark proceeded very fast with a high reaction rate constant—being 77 times higher compared with TC degradation by EDTA. The storage temperature of 30 °C contributed to a 4-fold increase in the *k* of Fe(III)-EDTA in the dark. In turn, in the case of samples stored with light access, the *k* constants determined for Fe(III)-EDTA were higher but still significantly lower than those determined for Fe(III)-Cit even in the dark. In the presence of Fe(III)-Cit and light, the TC degradation proceeds very fast, with *k* values 15 and 20 times higher than the values determined for Fe(III)-EDTA at 20 °C and 30 °C, respectively.

### 2.2. Fluorescence Spectra

**Figure 5:**
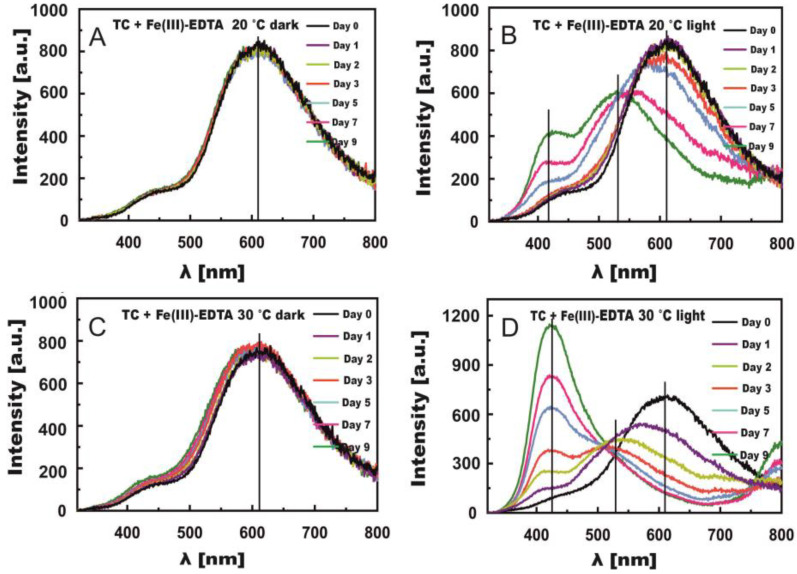
Changes in fluorescence spectra induced by Fe(III)-EDTA in TC solutions stored in the dark (**A**,**C**) and in light (**B**,**D**) at temperatures of 20 °C (**A**,**B**) and 30 °C (**C**,**D**).

The fluorescence spectra of TC with Fe(III)-EDTA and z Fe(III)-Cit were measured simultaneously with the absorption spectra. The measurements of TC + Fe(III)-EDTA revealed the formation of new TC fluorescence bands under the influence of Fe ions both at shorter wavelengths than the main maximum (ca. 610 nm) and at longer ones (Figure 5B,D).

Figure 5A–D present changes in the fluorescence spectra of TC solutions with Fe(III)-EDTA. Negligible changes could be observed in the spectra of the samples stored in the dark at both 20 °C and 30 °C, which fully confirms results obtained from absorption spectra. The irradiation of solutions caused new fluorescence bands to appear. For samples stored at 20 °C, the band at the max. wavelength of ca. 610 nm practically did not shift within the first 2 days (Figure 5B). A noticeable decrease in fluorescence intensity and band shift toward the shorter waves were observed already on day three. On day 7, the shift approximated 70 nm to λ = 550 nm, and on day 9 it reached 90 nm to λ = 530 nm. In addition, a new band appeared with the maximum at λ = 430 nm, which at 30 °C could be noticed as early as on day 5 and then became the major band on day 9. A new band also appeared above the wavelength of 800 nm, which was clearly noticeable on days 7 and 9 at 30 °C. Its presence was ascribed to the fluorescence of C-T complexes of TC with Fe(III)-Cit, which was signalled by Smyk et al. [36]. These complexes should form as the result of electron transfer from the benzene ring onto the quinone ring [48]. However, their formation requires the presence of a stacking complex formed by a nonoxidized and an oxidized molecule of TC. The presence of this complex is highly likely because the binding of the first Fe(III) ion is followed by TC oxidation and Fe(III) reduction to Fe(II) [36].

**Figure 6:**
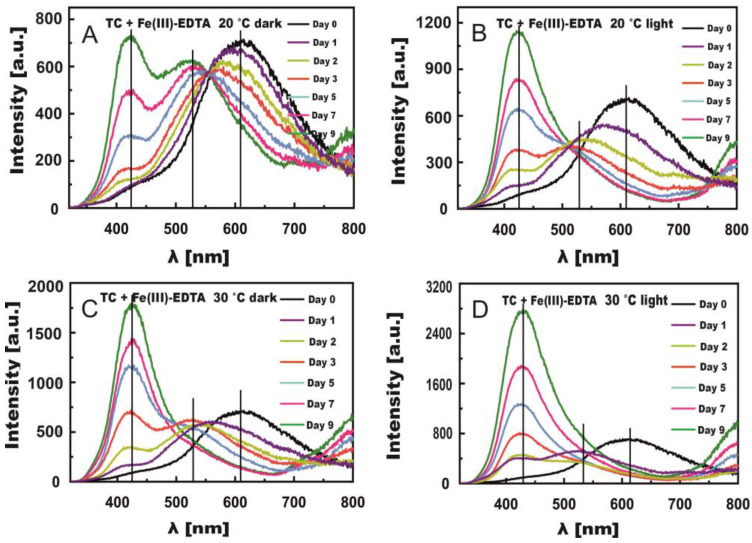
Changes in fluorescence spectra induced by Fe(III)-Cit in TC solutions stored in the dark (**A**,**C**) and in light (**B**,**D**) at temperatures of 20 °C (**A**,**B**) and 30 °C (**C**,**D**).

Figure 6A–D present changes in TC fluorescence spectra induced by Fe(III)-Cit for 9 days of measurements in the light and in the dark. The changes were remarkably greater than in the presence of Fe(III)-EDTA, even in the dark. The rate constants of this process were much higher in the presence of Fe(III)-Cit than Fe(III)-EDTA (Table 3). In the case of the samples stored at 20 °C, a large shift of the bands toward short waves was observed already after one day. After day five, 2 maxima were formed at λ = 430 nm and 530 nm, similar to the TC + Fe(III)-EDTA samples. At 30 °C, these 2 maxima appeared as early as on day two.

In light conditions, the reaction proceeded much faster compared with the reaction with Fe(III)-EDTA, especially at a temperature of 30 °C, where both maxima at λ = 430 nm and 530 nm could be observed after day one (Figure 6D). On day three, the predominating fluorescence band was formed with the maximum at λ = 430 nm. Its intensity reached the maximum value on day 9, which proves a high quantum yield of this band compared with the band with the maximum at λ = 530 nm (five-times higher fluorescence intensity of the band at 430 nm).

The appearance of new bands is indicative of the formation of TC complexes with Fe involving the attachment of Fe(III) ions to different sites on the TC ring. The binding of metal ions to a TC molecule is still being investigated. Various authors have suggested different sites of metal ion attachment to a TC molecule depending on the ion type. The binding sites can include C11 and C12 oxygen (or C10) of BCD rings. Metal ions may also attach to the N4 and O3 or C2 positions in the A ring [44,49,50,51,52].

The present study results indicate that band formation at 530 nm was due to the attachment of Fe(III) ions to the BCD ring of TC because it was associated with a simultaneously dropping band with maximum at λ = 610 nm. A wide TC band at λ = 610 nm was ascribed to proton transfer between carbon atoms C10-C12. Its disappearance along with the increasing concentration of Fe(III) ions in the solution proves that the binding of these ions displaces the proton at C11–O{H}O–C12 and eliminates the possibility of excited-state intramolecular proton transfer (ESIPT) [36,49]. Therefore, the attachment of metal ions was accompanied by the appearance of a new band at the wavelength of 530 nm. Schmitt et al. [50], who investigated the impact of magnesium and calcium ions on TC, noticed that the attachment of the first metal ion triggered the inhibition of proton transfer and that the second Mg^2+^ ion attached to the A ring of TC.

In our case, TC reaction with Fe(III) ions attaching to the BCD ring proceeded first, which points to the disappearance of the TC band at λ = 610 nm and the formation of a new band at λ = 530 nm. The course of TC reaction with Mg ions was similar [37]. These authors noticed the appearance of a new band for Mg with the maximum at 525 nm (in the present work, the band maximum for Fe ions was at λ = 530 nm).

The formation of the second band at 430 nm observed in our study could be due to the attachment of the second Fe(III) ion to the A ring of the TC molecule. Many authors have suggested the likelihood of metal ion attachment to the A ring of tetracycline [51,52,53].

Our previous research [37] demonstrated that Mg^+2^ ions attached to the A ring of tetracycline when their concentration in the solution was high. Because in the present study, we excited TC molecules with Fe(III) ions using the excitation wavelength of λ_exc_ = 300 nm, we also excited both the BCD and A chromophore of tetracycline. Given the above, it is likely that—as in the case of Mg ions [37]—the Fe ions also first displaced C11–O{H}O–C12 in the BCD ring, and then attached to the A ring under favorable conditions (light, temperature, reaction duration). In the case of Mg ions, this reaction was forced by their high concentration [37] and also by light, temperature, and reaction duration.

In our experiments, therefore, the absorption and fluorescence spectra of TC were modified by the type of compound (Fe(III)-EDTA and Fe(III)-citrate), light, temperature, and reaction duration. Notably, Fe(III) attachment to the A ring triggered the formation of a fluorescence band (at λ = 430 nm) with a high intensity compared with the band at 530 nm. Hence, the band at 530 nm was hidden under the band at 430 nm having a fivefold higher fluorescence intensity (Figure 6D).

**Figure 7:**
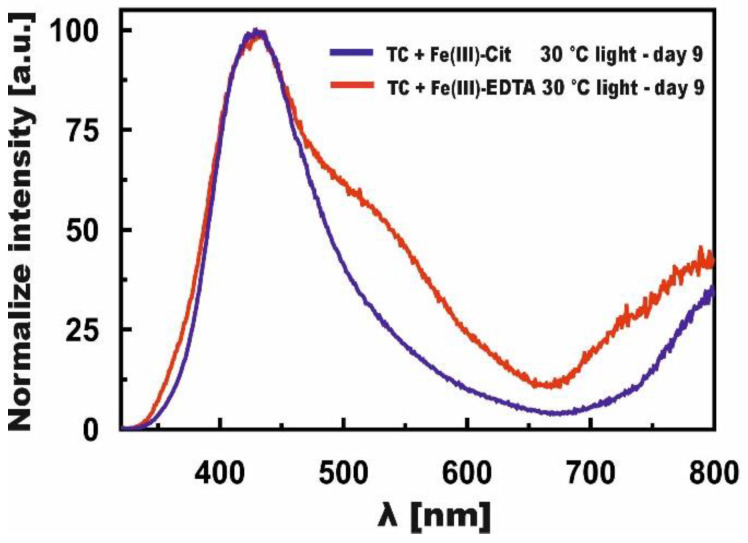
Normalized, final fluorescence spectra of TC in the presence of Fe(III)-Cit—blue line, and Fe(III)-EDTA—red line.

The normalized bands, presented in Figure 7, depict changes induced by Fe(III)-EDTA (curve day 9 from Figure 5D) and Fe(III)-Cit (curve day 9 from Figure 6D) in the TC samples stored in light at 30 °C, in the case of which the reaction rate constants were the highest. It can be seen from Figure 7 that the reactions proceeded faster for Fe(III)-Cit because in the case of Fe(III)-EDTA, there is still a visible band located at λ = 530 nm, which resulted from the iron ion joining the BCD ring. In both cases, the course of the reaction was similar because the bands at λ = 430 nm overlapped.

The small changes observed in the absorption and fluorescence spectra of TC with EDTA over 9 days at both 20 °C and 30 °C, compared with the significant changes observed for TC with Fe(III)-Cit, clearly indicate and confirm the prevailing role of ferric ions in TC degradation, which dissociated with Fe(III)-EDTA and Fe(III)-Cit and attached to TC. This degradation reaction was faster and more efficient upon the use of Fe(III)-Cit.

Jeong et al. [54] showed that oxidation and reduction may also contribute to a degradation of TC. They applied radiolysis of water, a method that produces highly reactive species (·OH, e^−^_aq_, and H·). The degradation efficiency was 40% after the application of ·OH and 23% in the case of e^−^_aq_. Our study shows that Fe binding to TC is an important stage in the degradation of this antibiotic; the degradation rate of TC by Fe^3+^ released from Fe(III)-EDTA at light was just 56%, while Fe^3+^ released from Fe(III)-Cit degraded as much as 86% of TC within the same period (three days—see Table 2). Various ways of the degradation, destruction or removal of TC have been described in the literature, including advanced oxidation processes (photolysis, ozonation and catalytic/UV light-based degradation), complexation of Fe(II)/(III) ions by TC and transformation of the antibiotic within the complex, membrane filtration, reverse osmosis and TC absorption/adsorption on various [ad]sorbents, including nanocomposites [44,54]. Iron can stimulate tetracycline degradation in at least two ways: indirectly by providing reactive oxygen species (ROS; released from water) or directly by joining tetracycline at specific sites of the molecule and forming unstable complexes with it. The second mechanism heavily relies on the availability of free iron ions. Therefore, we compared two iron complexes as sources of free Fe^3+^ ions. We assumed based on our previous studies and the literature that the critical role of both Fe(III)-EDTA and Fe(III)-Cit in tetracycline degradation is the release of Fe^3+^ cations rather than Cit or EDTA anions. We demonstrated and explained why Fe(III)-Cit releases Fe(III) more readily than the Fe(III)-EDTA does.

Tetracycline degradation can also be stimulated by photolysis, which generates singlet oxygen in the tetracycline solution [26], and this process is enhanced by iron ions provided in the form of FeCl_3_·6H_2_O. Our study clearly shows that Fe^3+^ ions derived from Fe(III)-Cit caused a 66% drop in tetracycline in darkness after as little as three days (Table 2). The result was confirmed by the fluorescence spectra in which new bands were detected (comp. Figure 6), corresponding to tetracycline with Fe bound at various sites. The TC degradation observed in previous papers [26,54] proceeded by the radiolysis of water and photolysis. In our experiments, the mechanism was different: The TC degradation was not caused by light but by the Fe^3+^ ions released from Fe(III)-EDTA and Fe(III)-Cit and bound directly to tetracycline rings BCD and A. Light did affect the process by releasing Fe^3+^ from Fe(III)-EDTA and Fe(III)-Cit. We have shown that Cit is able to degrade tetracycline even in darkness. We conclude therefore, that TC “pulls out” Fe ions from tetracycline even when there is no light, but favourable steric effects occur. This phenomenon practically did not occur in the case of EDTA because as we have shown, the energy of Fe^3+^ release from this compound is higher than from Cit.

In the case of Fe(III)-EDTA, light had a significant effect on TC degradation rate. In the case of Fe(III)-Cit, degradation reactions proceeded fast even in the dark, and k for Fe(III)-Cit in the dark at 20 °C was 77 times higher than k for TC degradation by Fe(III)-EDTA.

### 2.3. Quantum Chemistry Calculations

The observed changes in TC spectroscopic studies indicate difficulties in dissociating Fe ions from Fe(III)-EDTA and their easy release by dissociation from Fe(III)-Cit in the dark. In order to explain this phenomenon, we performed the quantum chemistry calculations of the free energy of iron release from both compounds. Molecules Fe(III)-EDTA and Fe(III)-Cit in the apo form exist in conformational equilibrium in solution. Table 4 (B3LYP) and Table 5 (BHANDHLYP) show the numbers of conformers predicted by GMMX software as well as important low-energy configurations with the largest input to the final Gibbs free energy of the ensemble of structures, which is shown in the last columns of both tables.

**Figure 8:**
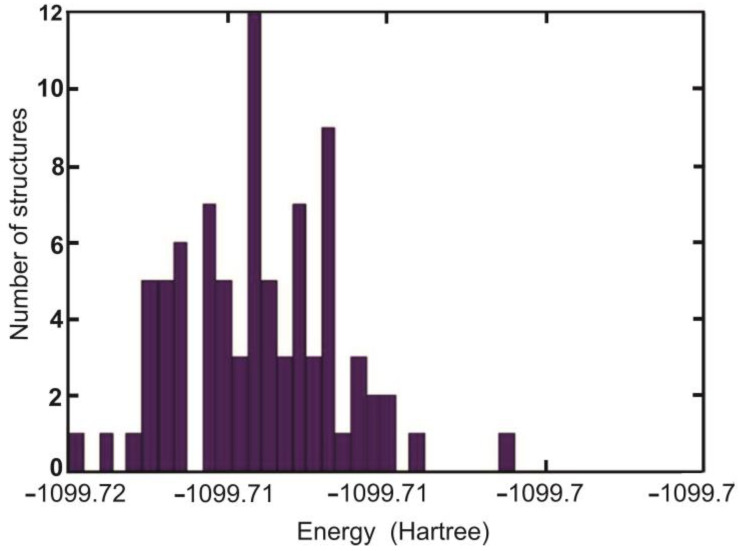
Energy distribution of EDTA apo structures optimized with BHANDHLYP/6-311 + G(d,p)/IEF-PCM method.

**Figure 9:**
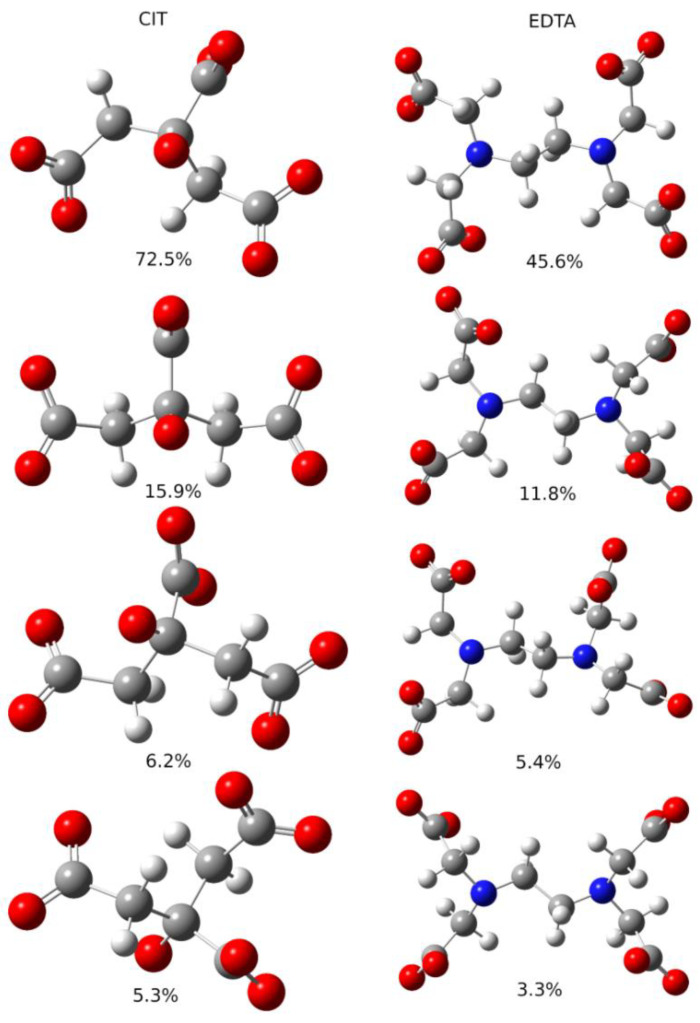
Most populated conformers of apo forms of Cit (left column) and EDTA (right column) molecules. Energies of molecules were calculated at BHANDHLYP/6-311 + G(d,p)/IEF-PCM level. Population of each conformer is given below its picture.

It was assumed that important configurations are those with population >1%. The energy distribution of apo-EDTA conformers is shown in Figure 8. The most populated conformers of Cit and EDTA are shown in Figure 9.

**Figure 10:**
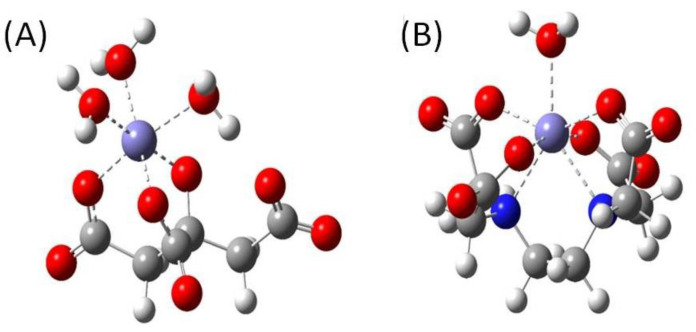
Optimized geometries of (**A**) Cit and (**B**) EDTA complexes with Fe(III) and water molecules. HANDHLYP/6-311 + G(d,p)/IEF-PCM method was applied.

Optimized conformations of Fe(III)-coordinating Cit and EDTA molecules are shown in Figure 10. Free energies of formation of Cit and EDTA complexes calculated with Equations (1) and (2) are −165.3 kcal/mol and −181.1 kJ/mol, respectively. Therefore, computations with B3LYP functional predict that complex of iron with EDTA is by 15.8 kcal/mol more stable than complex of iron with Cit molecule. Although the hybrid BHANDHLYP method predicts significantly lower free energies of formation of both molecules equal to −92.4 kcal/mol and −109.1 kcal/mol for Cit and EDTA molecules, respectively, the difference between free energies of formation is 16.7 kJ/mol in favour of EDTA molecules.

**Figure 11:**
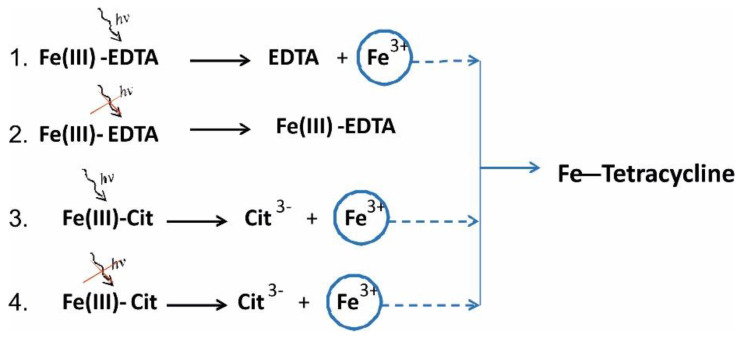
Proposed scheme of TC degradation under different lighting conditions. The degradation rate constants (k) of TC with Fe(III)-EDTA and Fe(III)-Cit, respectively, at 20 °C are following: (1) *k* = 1012 M^−1^day^−1^, (2) *k* = 55.4 M^−1^day^−1^, (3) *k* = 15,440 M^−1^day^−1^, (4) *k* = 4240 M^−1^day^−1^.

Therefore, both methods showed a significantly higher stability of EDTA-iron(III) complex than Cit-iron(III), with similar free energy difference. The obtained results explain why Fe ions from Fe(III)-EDTA molecules were released very poorly and did not degrade TC in the dark (Figure 11). Our absorption analysis results showed that only upon the activation of Fe(III)-EDTA by light did the complex release Fe ions that attached to TC and degraded it.

In solutions containing Fe(III)-Cit at equilibrium, more Fe ions are in the unbound state than in Fe(III)-EDTA solutions. Since there are more free ions, the probability of the degradation of the tetracycline molecule by the ion is higher, resulting in a higher degradation rate. Performed quantum chemistry calculations of the free energy of iron release from both compounds showed that the iron complex with EDTA is indeed more stable (free energy of the ensemble is 15.8 kcal/mol lower) than the iron complex with Cit; hence, Fe release from Fe(III)-EDTA is less effective.

## 3. Materials and Methods

Tetracycline hydrochloride (≥95%, Sigma-Aldrich, Poznań, Poland; TC) (Figure S1A), Fe(III) sodium ethylenediaminetetraacetate Na[Fe(EDTA)] × 3H_2_O (Fe(III)-EDTA) (Figure S1B) and Fe(III)-citrate (Fe(III)-Cit) (Figure S1C) were used. Fe(III)-EDTA and Fe(III)-Cit were prepared following the Resource Book for Sixth-form Practical Chemistry [55].

Two series of solutions were prepared: (1) TC solution C = 5 × 10^−5^ M with Fe(III)-EDTA C = 1 × 10^−4^ M; and (2) TC solution C = 5 × 10^−5^ M with Fe(III)-Cit C = 5 × 10^−5^ M, pH 5.6. The solutions were prepared using ultrapure water (Millipore Milli-Q^®^, Merck, Rahway, NJ, USA). These solutions were incubated for 9 days at 20 °C and 30 °C in darkness and in light using Philips Essential 14W/840 Cool White lamp (5.5 kLx illumination). The samples were taken out from the incubator only for the time of measurements. The first measurement (day 0) was performed one hour after mixing solution’s components. The absorption spectra were measured at 20 °C and 30 °C using the Cary 5000 spectrophotometer (Varian, Belrose, NSW, Australia) with a Peltier thermostated 1 cm cell holder. The TC concentrations were calculated from the absorbance at 353 nm, based on Lambert–Beer’s law.

Fluorescence was measured with a Cary Eclipse fluorescence spectrometer (Agilent, Melbourne, Australia) in a 1 cm quartz cell using the right-angle geometry. Excitation and emission slits were set at 20 nm. Instrument was equipped with a Peltier accessory. Temperature was stabilized at 20 °C and 30 °C. Fluorescence spectra were measured at λ_ex_ = 300 nm with cut-off filters: 360–1000 nm to avoid Raman peak of the solvent and the second-order peak generated by the grating. Every fluorescence spectrum was corrected for wavelength-dependent instrument sensitivity function and for inner filter effects I and II [56] using Grams AI Spectroscopy Software v. 9.2 (Thermo Fisher Scientific, Waltham, MA, USA). Therefore, intensities in different parts of the spectrum and between spectra can be compared quantitatively.

### Quantum Chemistry Calculations

The binding free energies for both Cit and EDTA complexes with iron were estimated using the following equations:

(1) $$\Delta G_{CIT}^{bind}=G_{CIT+Fe\left(III\right)+3H_{2}O}-\left(\langle G_{CIT}\rangle +G_{Fe\left(III\right)}+3G_{H_{2}O}+G_{CIT,dep}\right)$$

(2) $$\Delta G_{EDTA}^{bind}=G_{EDTA+Fe\left(III\right)+H_{2}O}-\left(\langle G_{EDTA}\rangle +G_{Fe\left(III\right)}+G_{H_{2}O}+G_{EDTA,dep}\right)$$

where $G_{CIT\left(EDTA\right)+Fe\left(III\right)}$ are Gibbs free energies of investigated molecules coordinating iron with their carboxyl and hydroxyl groups and water molecules, $\langle G_{CIT\left(EDTA\right)}\rangle \;$are ensemble averages of Gibbs free energies of formation of Cit(EDTA) molecules, $G_{Fe\left(III\right)}$ and $G_{H_{2}O}$ are Gibbs free energies of iron and water, respectively. It was assumed that Fe(III) is coordinated by two carboxyl groups, one hydroxyl group of single Cit molecule and three water molecules, and therefore its coordination number is six. In EDTA, it was assumed that Fe(III) is coordinated by four carboxyl groups, two nitrogens and one water molecule, and therefore its coordination number is seven. $G_{CIT\left(EDTA\right),dep}$ are free energies of deprotonation of both molecules calculated from experimentally determined pK’a values: 0, 1.5, 2.0, 2.66 (carboxyl groups) and 6.16, 10.24 (amino groups) for EDTA molecule [57] and 3.13, 4.76, 6.4 (carboxyl groups) and 14.4 (hydroxyl group) for Cit molecule [58,59].

All free energies were calculated with DFT using B3LYP functional [60] and 6-311+G(d,p) basis set and SDD semi-relativistic pseudo-potentials applied to Fe(III) [61] with implicit water solution emulated by IFE-PCM model [62,63]. Free energies were also estimated using hybrid BHANDHLYP [64] with the same basis set and solvation model. Geometries of all initial structures were optimized followed by calculation of oscillation frequencies, which were used for free energy corrections. No imaginary frequencies were observed, proving that each structure reached energy minimum. Ensembles of Cit(EDTA) molecules in apo forms were determined using conformer-search tool (GMMX) of Gaussian software, using classical mechanics UFF force-field [65]. The initial structures obtained with GMMX were then optimized with B3LYP/6-311+G(d,p)/IEF-PCM and BHANDHLYP/6-311+G(d,p)/IEF-PCM methods in order to determine final Gibbs free energy of ensemble of Cit(EDTA) structures. It was assumed that multiplicity of both free and bound Fe(III) is equal to six.

## 4. Conclusions

Our experiments have shown that Fe(III)-Cit is much more efficient in inducing the tetracycline breakdown compared with Fe(III)-EDTA because iron is more strongly bound with EDTA than Cit. The detachment of iron from EDTA does occur, but it requires light. The changes in the absorption spectra of TC solutions with Fe(III)-EDTA stored in the dark at both 20 °C and 30 °C were negligible. It was only under light conditions that a 50% drop in long wave absorption band of TC was observed. Tetracycline degradation proceeds through binding of iron released from these compounds. The rate constants of tetracycline degradation determined in darkness were 77 and 50 times higher for the Fe(III)-Cit compared to Fe(III)-EDTA samples stored at 20 °C and 30 °C, respectively. The rapid TC degradation caused by Fe(III)-Cit could be the result of its lower thermodynamic stability compared with Fe(III)-EDTA. The above results were confirmed by two quantum chemistry calculations that showed that the iron complex with EDTA is indeed more stable (free energy of the ensemble is 15.8 kcal/mol lower) compared with the iron complex with Cit, hence Fe release from Fe(III)-EDTA is less effective.

## Figures and Tables

**Table 1:** Tetracycline concentrations [M] and percent ratios of TC concentrations in samples treated with Fe(III)-EDTA relative to controls for incubations at darkness and light, at 20 °C and 30 °C.

Days	TC with Fe(III)-EDTA 20 °C				TC wtih Fe(III)-EDTA 30 °C			
	Dark		Light		Dark		Light	
	C[M] ± SD × 10^−5^	%	C[M] ± SD × 10^−5^	%	C[M] ± SD × 10^−5^	%	C[M] ± SD × 10^−5^	%
0	5.00 ± 0.02	100 ± 0	5.00 ± 0.02	100 ± 0	4.97 ± 0.01	99 ± 0	4.97 ± 0.01	99 ± 1
1	4.95 ± 0.05	99 ± 1	4.86 ±0.10	97 ± 2	4.89 ± 0.05	98 ± 1	4.70 ± 0.10	90 ± 2
2	4.92 ± 0.10	98±2	4.67 ± 0.10	94 ± 2	4.83 ± 0.10	97 ± 2	4.20 ± 0.19	84 ± 4
3	4.92 ± 0.10	98±2	4.48 ± 0.15	90 ± 3	4.79 ± 0.10	96 ± 2	3.83 ± 0.15	77 ± 3
5	4.89 ± 0.05	98 ± 1	4.00 ±0.15	80 ± 3	4.66 ± 0.05	94 ± 1	2.19 ± 0.25	44 ± 5
7	4.86 ± 0.05	97 ± 2	*	*	4.57 ± 0.10	92 ± 2	*	*
9	4.85 ± 0.05	97 ± 3	*	*	4.52 ± 0.15	91 ± 3	*	*

* absorbance increase.

**Table 2:** Tetracycline (TC) concentration [M] and percent ratio of TC concentration in a solution supplemented with Fe(III)-Cit relative to control for samples stored in darkness or light at 20 °C and 30 °C.

Days	TC with Fe(III)-Cit 20 °C				TC with Fe(III)-Cit 30 °C			
	Dark		Light		Dark		Light	
	C[M] ± SD × 10^−5^	%	C[M] ± SD × 10^−5^	%	C[M] ± SD × 10^−5^	%	C[M] ± SD × 10^−5^	%
0	4.26 ± 0.01	85 ± 2	4.26 ± 0.01	85 ± 3	4.17 ± 0.02	83 ± 1	4.17 ± 0.02	83 ± 1
1	3.86 ± 0.13	77 ± 3	3.19 ± 0.17	64 ± 4	2.94 ± 0.13	59 ± 3	1.47 ± 0.08	29 ± 2
2	3.43 ± 0.08	69 ± 2	2.36 ± 0.08	47 ± 2	2.09 ± 0.08	42 ± 2	0.82 ± 0.24	16 ± 4
3	2.96 ± 0.17	59 ± 4	1.47 ± 0.04	29 ± 1	1.71 ± 0.04	34 ± 1	0.71 ± 0.12	14 ± 3
5	2.32 ± 0.21	45 ± 3	1.03 ± 0.05	21 ± 2	1.25 ± 0.04	25 ± 1	*	*
7	1.86 ± 0.17	37 ± 4	*	*	*	*	*	*
9	1.69 ± 0.13	34 ± 3	*	*	*	*	*	*

* absorbance increase.

**Table 3:** The reaction rate constants for TC degradation under different experimental conditions (light or darkness, 20 °C and 30 °C temperature) in the presence of Fe(III)-EDTA or Fe(III)-Cit.

	Rate Constant			
	Dark		Light	
	20 °C	30 °C	20 °C	30 °C
Fe(III)-EDTA				
*k* [M^−1^day^−1^]	55.4 ± 6.8	227 ± 12	1012 ± 93	2050 ± 210
R^2^	0.9296	0.9862	0.9751	0.9787
Fe(III)-Cit				
*k* [M^−1^day^−1^]	4240 ± 180	11,330 ± 290	15,440 ± 1450	40,270 ± 5180
R^2^	0.9913	0.9980	0.9744	0.9679

**Table 4:** Properties of all molecules calculated with B3LYP/6-311 + G(d,p)/IEF-PCM method.

Molecule	Number of Conformers Obtained from GMMX	Number of Conformers Successfully Optimized with QM Method	Number of Important Low-Energy Conformations (Population > 1%)	Energies of Low-Energy Conformations (Hartree)	Free Energy of the Ensemble (Hartree)
Cit	5	4	4	−758.350139	−758.350832
				−758.349923	
				−758.348538	
				−758.348006	
EDTA	132	100 (−4 duplicates)	15	−1100.360670	−1100.361119
				−1100.360201	
				−1100.358954	
				−1100.358872	
Fe(III)	1	1	1	−123.298870	−123.298870
H_2_O	1	1	1	−76.462870	−76.462870
Cit+Fe(III) + 3H_2_O	1	1	1	−1111.363139	−1111.363139
EDTA+Fe(III) + H_2_O	1	1	1	−1300.459440	−1300.459440

**Table 5:** Properties of all molecules calculated with BHANDHLYP/6-311 + G(d,p)/IEF-PCM method.

Molecule	Number of Conformers Obtained from GMMX	Number of Conformers Successfully Optimized with QM Method	Number of Important Low-Energy Conformations (Population > 1%)	Energies of Low-Energy Conformations (Hartree)	Free Energy of the Ensemble (Hartree)
Cit	5	4	4	−757.917685	−757.918345
				−757.917232	
				−757.916617	
				−757.915169	
EDTA	132	83 (−1 duplicate)	19	−1099.719945	−1099.720685
				−1099.718671	
				−1099.717932	
				−1099.717467	
Fe(III)	1	1	1	−123.298870	−123.298870
H_2_O	1	1	1	−76.418013	−76.418013
Cit + Fe(III) + 3H_2_O	1	1	1	−1110.680331	−1110.680331
EDTA + Fe(III) + H_2_O	1	1	1	−1299.659800	−1299.659800

## Data Availability

Not applicable.

## Supplementary Materials

The following supporting information can be downloaded at: www.mdpi.com/article/10.3390/molecules27238498/s1, Figure S1: Structure of tetracycline (A), structure of seven-coordinate mono-aqua Fe(III)(EDTA)(H_2_O)]^−^ complex (B), and Fe(III)-citrate (C).
