# Supplementary material for: Studies on the Efficiency of Iron Release from Fe(III)-EDTA and Fe(III)-Cit and the Suitability of These Compounds for Tetracycline Degradation
Source: Molecules. 2022 Dec 2;27(23):8498. doi: 10.3390/molecules27238498 (PMC9739602; doi:10.3390/molecules27238498)
Supplement: Supplementary file 1 [file molecules-27-08498-s001.zip › molecules-2057480-supplementary.pdf]

Studies on the Efficiency of Iron Release from Fe(III)-EDTA and Fe(III)-Cit and the Suitability of These Compounds for Tetracycline Degradation

Agnieszka I. Piotrowicz-Cieślak [1,*], Maciej Maciejczyk [2], Małgorzata Margas [1], Dariusz Rydzyński [1], Hanna Grajek [2], Dariusz J. Michalczyk [1], Janusz Wasilewski [3] and Bogdan Smyk [2]

[1] Department of Plant Physiology, Genetics and Biotechnology, Faculty of Biology and Biotechnology, University of Warmia and Mazury in Olsztyn, Oczapowskiego 1A, 10-718 Olsztyn, Poland
[2] Department of Physics and Biophysics, Faculty of Food Science, University of Warmia and Mazury in Olsztyn, Oczapowskiego 4, 10-719 Olsztyn, Poland
[3] Department of Biochemistry, Faculty of Biology and Biotechnology, University of Warmia and Mazury in Olsztyn, Oczapowskiego 1A, 10-718 Olsztyn, Poland
* Correspondence: acieslak@uwm.edu.pl

SUPPLEMENTARY

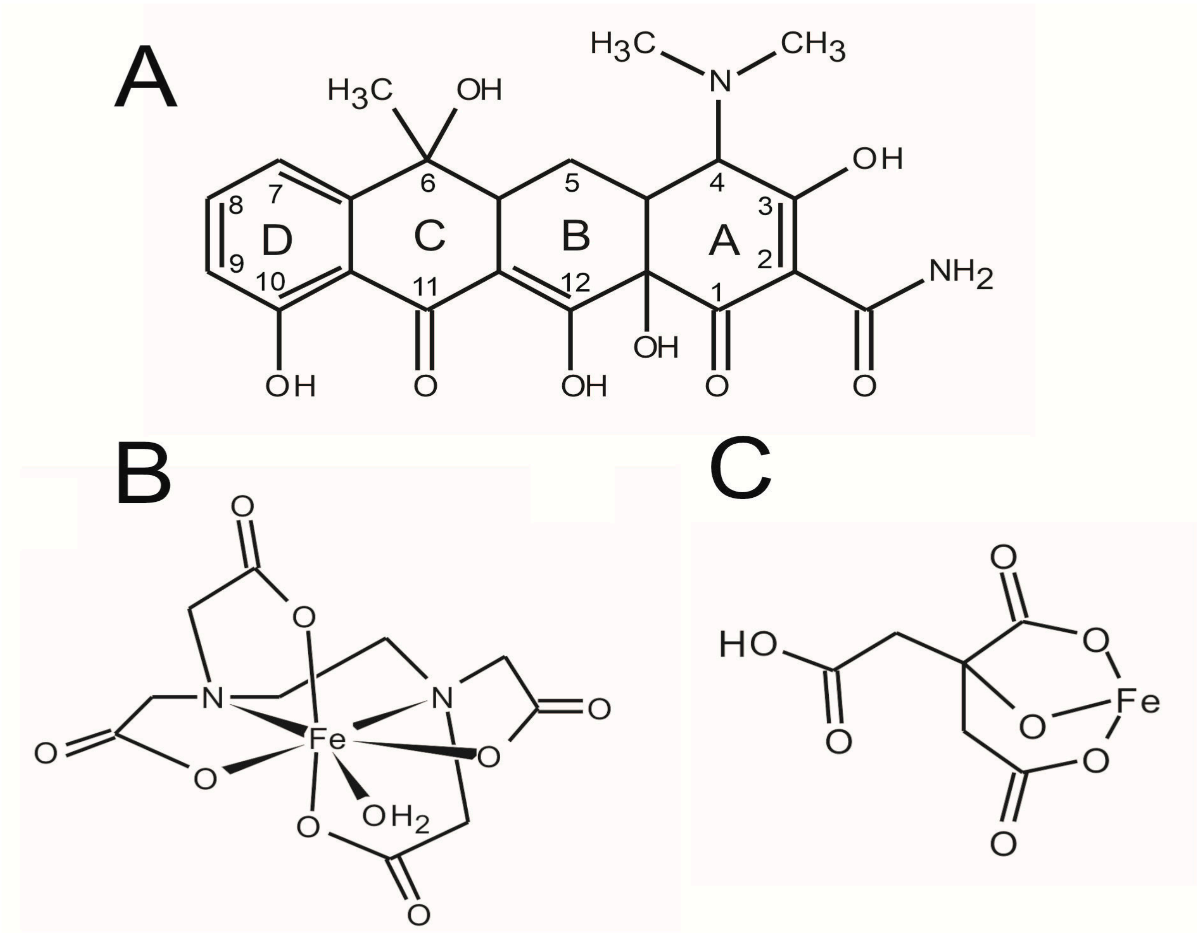

Figure S1. Structure of tetracycline (A), structure of seven-coordinate mono-aqua $Fe(III)(EDTA)(H_2O)]^-$ complex (B), and Fe(III)-citrate (C).
